# Mge-cluster: a reference-free approach for typing bacterial plasmids

**DOI:** 10.1093/nargab/lqad066

**Published:** 2023-07-10

**Authors:** Sergio Arredondo-Alonso, Rebecca A Gladstone, Anna K Pöntinen, João A Gama, Anita C Schürch, Val F Lanza, Pål Jarle Johnsen, Ørjan Samuelsen, Gerry Tonkin-Hill, Jukka Corander

**Affiliations:** Department of Biostatistics, University of Oslo, Oslo, Norway; Parasites and Microbes, Wellcome Sanger Institute, Cambridge, UK; Department of Biostatistics, University of Oslo, Oslo, Norway; Department of Biostatistics, University of Oslo, Oslo, Norway; Norwegian National Advisory Unit on Detection of Antimicrobial Resistance, Department of Microbiology and Infection Control, University Hospital of North Norway, Tromsø, Norway; Department of Pharmacy, Faculty of Health Sciences, UiT The Arctic University of Norway, Tromsø, Norway; Department of Medical Microbiology, UMC Utrecht, Utrecht, The Netherlands; CIBERINFEC, Madrid, Spain; Bioinformatics Unit, University Hospital Ramón y Cajal, IRYCIS, Madrid, Spain; Department of Pharmacy, Faculty of Health Sciences, UiT The Arctic University of Norway, Tromsø, Norway; Norwegian National Advisory Unit on Detection of Antimicrobial Resistance, Department of Microbiology and Infection Control, University Hospital of North Norway, Tromsø, Norway; Department of Pharmacy, Faculty of Health Sciences, UiT The Arctic University of Norway, Tromsø, Norway; Department of Biostatistics, University of Oslo, Oslo, Norway; Parasites and Microbes, Wellcome Sanger Institute, Cambridge, UK; Department of Biostatistics, University of Oslo, Oslo, Norway; Parasites and Microbes, Wellcome Sanger Institute, Cambridge, UK; Department of Mathematics and Statistics, Helsinki Institute of Information Technology (HIIT), FI-00014 University of Helsinki, Helsinki, Finland

## Abstract

Extrachromosomal elements of bacterial cells such as plasmids are notorious for their importance in evolution and adaptation to changing ecology. However, high-resolution population-wide analysis of plasmids has only become accessible recently with the advent of scalable long-read sequencing technology. Current typing methods for the classification of plasmids remain limited in their scope which motivated us to develop a computationally efficient approach to simultaneously recognize novel types and classify plasmids into previously identified groups. Here, we introduce *mge-cluster* that can easily handle thousands of input sequences which are compressed using a unitig representation in a de Bruijn graph. Our approach offers a faster runtime than existing algorithms, with moderate memory usage, and enables an intuitive visualization, classification and clustering scheme that users can explore interactively within a single framework. *Mge-cluster* platform for plasmid analysis can be easily distributed and replicated, enabling a consistent labelling of plasmids across past, present, and future sequence collections. We underscore the advantages of our approach by analysing a population-wide plasmid data set obtained from the opportunistic pathogen *Escherichia coli*, studying the prevalence of the colistin resistance gene *mcr-1.1* within the plasmid population, and describing an instance of resistance plasmid transmission within a hospital environment.

## INTRODUCTION

Bacteria can exchange genetic material via Horizontal Gene Transfer (HGT) mediated by Mobile Genetic Elements (MGEs) such as temperate phages and plasmids. Plasmids act as key vehicles for the dissemination of important traits such as antimicrobial resistance (AMR) and virulence both within and between species (1,2). The introduction and broad implementation of long-read sequencing for the assembly of bacterial genomes have led to a dramatic increase in the number of complete plasmid sequences (3).

Clustering and classifying complete plasmid sequences into meaningful groups is a crucial step to understanding the epidemiology of plasmid-encoded genes (4). Without a consistent plasmid typing scheme, it is challenging to examine, for example, whether AMR genes are disseminated by a single or several plasmid types, or if particular plasmid types are overrepresented in successful bacterial clones. Current plasmid typing tools struggle to account for the extreme modularity observed in plasmids, where large genomic blocks can be rapidly gained or lost. Traditionally, plasmids have been classified according to their replicon and associated incompatibility (Inc) groups using tools such as PlasmidFinder (5,6). However, replicon-based typing suffers from the presence of multiple replicons within the same sequence, offers a limited resolution for epidemiological purposes (4) and is only well-established in particular bacteria phyla (e.g. Proteobacteria). Another strategy consists of typing plasmids based on their relaxase, a protein involved in plasmid mobilisation (7,8), which is in turn limited to plasmids transmissible by conjugation.

Network analyses based on k-mers or average nucleotide identities (ANI) have been proposed as an alternative classification framework (9,10). This strategy was implemented in the recent release of COPLA, a novel tool to classify sequences into discrete plasmid taxonomic units (PTUs) based on ANI distances and hierarchical stochastic block modelling (11). MOB-suite is another tool that classifies sequences but relies on k-mers observed in the entire plasmid (12,13). MOB-suite uses Mash distances coupled with complete linkage clustering to partition plasmid sequences by maximising consistency with replicon and relaxase schemes. The use of COPLA is mainly restricted to typing small sets of sequences due to its computation-intensive algorithm while MOB-suite is more scalable. MOB-suite uses a single Mash threshold to cluster plasmid sequences into discrete groups and can fail to accurately cluster collections of MGEs with different sequence sizes or gene gain/loss rates.

Here, we present mge-cluster, a novel approach to consistently type and classify MGEs. Mge-cluster provides a classification framework that allows for the typing of thousands of input sequences with a runtime faster than existing algorithms and moderate memory usage. Furthermore, in the light of new MGE data, it offers an option to type these new sequences with an existing mge-cluster model and avoids the need to reanalyse previously typed sequences. Mge-cluster considers the entire sequence content by extracting the unitig sequences which are extended nodes (k-mers) in a compressed de Bruijn graph. Briefly, unitigs correspond to the merging of all maximal-non-branching paths present in the compressed de Bruijn graph with a word size exceeding the original k-mer size (14). The presence/absence of unitigs is embedded into a 2D-representation using openTSNE (15–17), a non-linear dimensionality reduction algorithm that permits the addition of new points to an existing embedding. The non-linear aspect of the t-SNE algorithm allows for plasmid clusters to be identified at multiple scales of genetic variation. The HDBSCAN clustering algorithm is then finally used to define plasmid clusters in the resulting 2D embedding (18).

We demonstrate the features of mge-cluster by generating a plasmid classification framework for the opportunistic pathogen *Escherichia coli*, one of the leading causes of bloodstream and urinary tract infections globally with a large number of complete plasmid sequences available. In this organism, virulence factors are usually associated with plasmids, which drive the virulence of enteroinvasive, enteropathogenic, enterohemorrhagic, enteroaggregative, and extraintestinal pathogenic *E. coli* (19,20). Moreover, plasmids are key hosts for AMR determinants such as extended-spectrum β-lactamases and mobile colistin resistance genes contributing to the emergence of *E. coli* multi-drug resistant infections. Overall, mge-cluster provides a fast and consistent classification framework for MGEs that can be easily distributed to enhance the analysis and tracking of these elements.

## MATERIALS AND METHODS

### Overview

**Figure 1.**
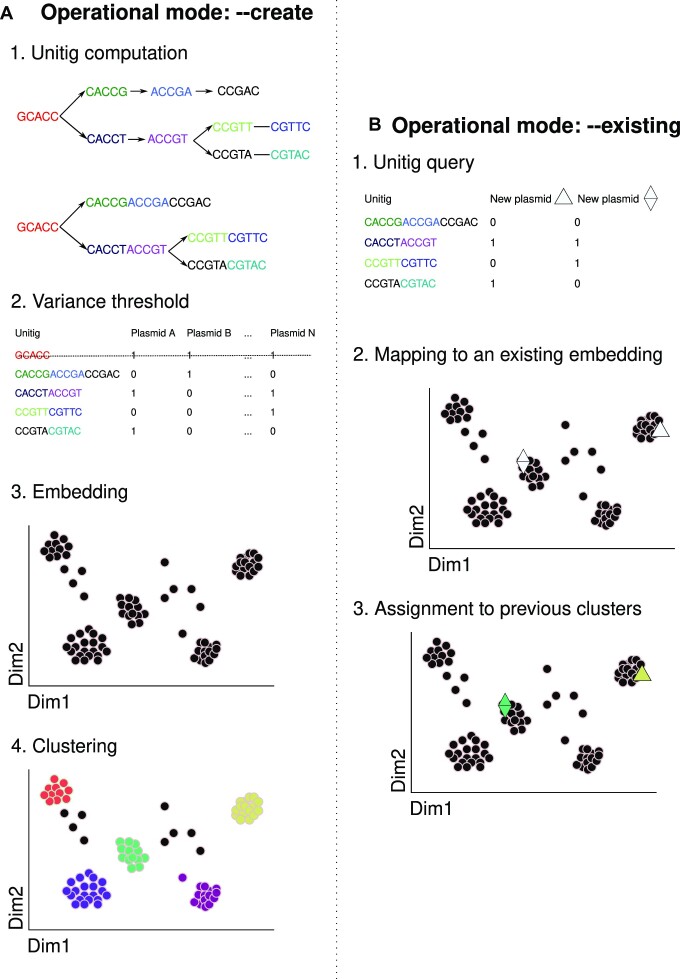
Graphical overview of the mge-cluster pipeline. (**A**) Schematic steps of the operational mode *–create* used by mge-cluster to define discrete cluster groups from a set of MGEs. (i) Unitigs are computed with ‘unitig-caller’ a wrapper script for the Bifrost algorithm (14). For graphical purposes, a k-mer size of 5 was considered to exemplify and simplify the unitig concept and reverse-complements were omitted. (ii) Unitigs with low variance in their presence and absence between samples are removed. (iii) The filtered unitig presence/absence matrix is then transformed into Jaccard distances and embedded into 2 dimensions using openTSNE (16). (iv) HDBSCAN (18) is used to call clusters in the 2D embedding defined by openTSNE. In this example, five different clusters (highlighted with distinct colours) are represented while points in black correspond to unassigned sequences. (**B**) Schematic steps of the operational mode –*existing* used by mge-cluster to assign to a new set of MGEs with the same clusters computed in a previous model. (i) The same selected unitig definitions present in the model are queried to create the classification features. (ii) openTSNE maps the new samples to the existing embedding. (iii) HDBSCAN assigns the new samples to a previously defined cluster.

Mge-cluster is an open-source approach to cluster and classify MGEs. The tool accepts assembled nucleotide sequences for a set of MGEs as input. To generate initial clusters, mge-cluster uses the –*create* operational mode (Figure 1A). Mge-cluster identifies all unitigs in the set of MGEs (default k-mer 31) by generating a de Bruijn graph using Bifrost (14). Unitigs with a low variance in their presence and absence between samples are filtered out. The remaining unitigs are used as classification features and pairwise Jaccard distances are calculated from the unitig presence/absence matrix. The resulting distance matrix is embedded into two dimensions using openTSNE (16) and HDBSCAN is used to identify clusters in the 2D embedding (18). For each sample, mge-cluster returns the 2D embedding coordinates and the assigned HDBSCAN cluster.

The classification mode of mge-cluster facilitates the contextualization of MGEs across distinct collections without the need to reanalyse the original dataset (Figure 1B). In this mode, mge-cluster computes the same unitig features as in the existing model. These unitigs are used by openTSNE and HDBSCAN to map the new samples into the existing 2D embedding and assign them to the previously defined clusters. The ability of mge-cluster to rapidly type MGEs enhances our capacity to compare MGEs between datasets and to conduct genomic surveillance of these important elements. A detailed description of the steps performed by mge-cluster is given in the sections below.

### Workflow

Mge-cluster is a Python package installable through bioconda https://anaconda.org/bioconda/mge-cluster, freely available under the open-source MIT license https://gitlab.com/sirarredondo/mge-cluster. Supplementary Figure S1 illustrates the two different operational modes of mge-cluster: *–create* and *–existing*. In both cases, mge-cluster takes as input a file that indicates the absolute or relative paths to the nucleotide sequence files (.fasta format). The *–create* mode will generate a new classification scheme for the sequences provided as input by the user while the *–existing* mode will return embedding coordinates and cluster assignments considering a previous, existing mge-cluster model. Both modes can be run with the multithreading option (*–threads*) to reduce mge-cluster runtime.

### Unitigs as classification features

Unitigs defined as extended nodes in a compressed de Bruijn graph were selected as features for building the classification framework. Unitig-caller (*–call* mode, version 1.2.1) https://github.com/bacpop/unitig-caller which implements Bifrost Build (14) is used with a k-mer size specified by the mge-cluster (argument *–kmer*) to generate a presence/absence matrix of the unitigs present in the input file.

Bifrost initially considers a *de Bruijn* graph structure defined as a direct graph:


$$\begin{equation*}G = ( {V,E} )\end{equation*}$$

*V* corresponds to the number of vertices (k-mers) present and *E* to the edges connecting the distinct vertices. Thus, the vertices *V* present in graph *G* can be defined by:


$$\begin{equation*}V\ = \ \left\{ {{v}_1,{v}_2,\ \ldots ,\ {v}_n} \right\}\end{equation*}$$

The edges *E* can be defined as direct connections between two vertices of *V*:


$$\begin{equation*}E\ = \ {\rm{\{ }}\left( {i,j} \right):{\rm{|}}1\ \le \ i,\ j\ \le \ n\ with\ an\ edge\ from\ {v}_i\ to\ {v}_j\} \end{equation*}$$

For each $v\ \epsilon \ V$, we define the in-degree ${d}^i( v )$ and out-degree ${d}^o( v )$ as the number of edges in *E* towards and from *v* respectively.

Paths in the graph can be defined as finite sequences of distinct vertices connected by edges $p\ = \ ( {{v}_o,\ e( {{v}_0,{v}_1} ),\ {v}_1,\ e( {{v}_1,{v}_2} ),\ \ldots ,\ e( {{v}_{k - 1},{v}_k} ),\ {v}_k} )$. Bifrost then considers all non-branching paths, defined as paths *p* in which all vertices have an ${d}^i( v )\ = \ 1$

and ${d}^o( v )\ = \ 1$ excluding the first and last vertices in *p*.

Each non-branching path is merged into a single vertex, termed unitig in Bifrost. Those unitigs represent extensions of the initial k-mers (vertices) that are longer in length than the original k-mer size. Unitig-caller then creates a presence/absence matrix of those unitigs. We can define *M*, as a binary matrix with $s * \mu$ dimensions, in which *s* is the total number of sequences present in the input file and *u* corresponds to the total number of unitigs extracted by Bifrost.


$$\begin{equation*} {\rm{\ }}{M}_{s,u} = \left( {\begin{array}{@{}*{4}{c}@{}} {{m}_{1,1}}&{{m}_{1,2}}& \ldots &{{m}_{1,u}}\\ {{m}_{2,1}}&{{m}_{2,2}}& \cdots &{{m}_{2,u}}\\ \vdots & \vdots & \ddots & \vdots \\ {{m}_{s,1}}&{{m}_{s,2}}& \cdots &{{m}_{s,u}} \end{array}} \right)\ \end{equation*}$$

Unitigs were chosen over other features (e.g. gene presence/absence) because identical unitig definitions could be computed between distinct datasets, an essential characteristic for the *–existing* prediction mode of mge-cluster. To reduce the memory use required to build the typing scheme, we remove unitigs with a variance less than 0.01 (default) using the function VarianceThreshold of the python package sklearn (version 1.0.2) (21). In this manner, we remove unitigs (features in the model) that have the same value for all samples and thus do not provide any relevant information for the embedding process. This variance threshold can be modified by the user in mge-cluster (argument *–variance*).

### Embedding the presence/absence of unitigs into a lower number of dimensions

We considered the implementation of the t-SNE algorithm available in the python package openTSNE (version 0.6.1) (16,17) to generate a 2D embedding based on *M*, the unitigs presence/absence matrix. This new implementation improved the global positioning of the points and introduced the possibility of mapping new points into an existing, reference embedding. The multidimensional presence/absence matrix of unitig-caller can be represented as $M\ = \ \{ {{m}_1,\ {m}_2,\ \ldots ,\ {m}_s} \}\ \epsilon \ {R}^u$ for which ${m}_s$ corresponds to a datapoint (sequence) with *u* defined as the number of dimensions (number of unitigs passing the variance threshold). In our case, openTSNE is run to find a 2D dimensional embedding $Y\ = \ \{ {{y}_1,\ {y}_2,\ \ldots ,\ {y}_s} \}\ \epsilon \ {R}^{2\ }$in which the original distance between ${m}_1$ and ${m}_s$ is preserved in ${y}_1$ and ${y}_s$. The similarity between two data points in the original space is measured with Jaccard distances (flag –*metric*). The perplexity value is one of the main parameters of openTSNE that affects how the similarity between two data points in the original space is preserved in the resulting embedding space. Large perplexity values tend to preserve the global structure of the data better while obscuring some of the local structure potentially resulting in small clusters being merged together. Small perplexity values generate tight dense clusters preserving the local structure better but ignoring the overall global structure for which the distance and position of the clusters in the resulting embedding can no longer be considered.

The TSNE function can be run with different perplexity values specified by the user with the mge-cluster arguments *–perplexity*, using ‘exact’ as the method for finding the nearest neighbor (flag *–neighbors*). For reproducibility purposes, we fixed the seed of the random number generator with the flag *–random_state*.

### Calling plasmid clusters in the embedding space

To define which clusters were present in the embedding space $Y\ = \ \{ {{y}_1,{y}_2,\ \ldots ,\ {y}_s} \}\ \epsilon \ {R}^2$ created by openTSNE, we required a clustering algorithm that (i) did not force us to provide the number of clusters present in the data, (ii) tolerated noisy data since plasmid modularity can result in sequences that are hybrids between two neighbouring clusters, (iii) tolerated clusters with different density and sizes (iv) allowed the assignment of new data points to an existing clustering solution. Based on these four premises, we selected the HDBSCAN algorithm (18), an improved version of dbscan that finds highly stable clusters over a range of epsilon values (the main parameter of dbscan).

HDBSCAN defines the mutual reachability distance (extracted from HDBSCAN documentation) as ${d}_{mreach - k}( {{y}_1,\ {y}_s} )\ = \ max( {cor{e}_k( {{y}_1} ),\ cor{e}_k( {{y}_s} ),\ d( {{y}_1,{y}_s} )} ),$ where $d( {{y}_1,{y}_s} )$ is the original metric distance (Euclidean) and $cor{e}_k$ the distance to its *k*th neighbour. This *mreach* distance is used to transform the embedding space into a new space where points with low core distances remain together while pushing away sparser points. This distance is considered to create a graph structure $HG\ = \ ( {P,D} )$ in which nodes *P* correspond to the original data points ${y}_s$ while *D* are all edges with weight equal to ${d}_{mreach - k}( {{y}_1,{y}_s} )$. HDBSCAN then transforms *HG* into a minimum spanning tree to look into the hierarchy of connected components. The parameter –*min_cluster_size* is used define the minimum number of points required to define a cluster. This parameter is then used to generate a condensed tree to select clusters with high persistence. Lastly, HDBSCAN outputs for each point ${y}_s$ their assigned cluster and membership probability.

The python package hdbscan (version 0.8.28) with the primary function HDBSCAN is run to specify a default minimum cluster size (flag *–min_cluster_size*) defined by the user in mge-cluster (argument *–min_cluster*).

The main output of this operational mode consists of a comma-separated file (csv) with the embedding coordinates given by openTSNE (columns ‘tsne1D’, ‘tsne2D’), the cluster assigned and membership probability returned by HDBSCAN (column ‘Standard_Cluster’ and ‘Membership_Probability’ and the last column (‘Sample_Name’) indicating the header extracted from the given nucleotide sequences.

### Storing and distributing an mge-cluster model

Mge-cluster was specifically designed to generate a classification scheme that can easily be distributed and reused by other users. The following files constitute a mge-cluster model: (i) *.unitigs.fasta, the fasta file containing the unitigs with a variance higher than specified in the argument *–variance*, (ii) *.embedding.pbz2, embedding model created by openTSNE to transform the unitig presence/absence matrix into 2D and (iii) *clusters.pbz2, clustering model created by HDBSCAN to call clusters in the resulting embedding from openTSNE.

### Prediction of a new batch of sequences using an existing mge-cluster model

For predicting the embedding coordinates and the cluster assignment of a new batch of plasmid sequences with an existing mge-cluster model, we designed the *–existing* operational mode (Figure 1B). In this mode, mge-cluster requires an input file pointing to the nucleotide sequences of interest and the folder with the files constituting a mge-cluster model (Supplementary Figure S1).

Mge-cluster performs the following steps: (i) computes the same unitig definitions present in the file *unitigs.fasta, using unitig-caller (–*query* mode), (ii) uses the transform function from openTSNE python package to embed the new points to the existing embedding present in the file *embedding.pbz2, (iii) assigns the new points to the existing HDBSCAN clusters present in the file *clusters.pbz2 using the approximate_predict function from the hdbscan python package.

### Benchmarking mge-cluster with real data: generating an *E. coli* model to classify plasmid sequences

To showcase mge-cluster on real data, we developed an *E. coli* model to classify plasmid sequences. We considered all plasmid sequences (*n* = 6864) with the species ‘*Escherichia coli’* annotated in the PLSDB database (22). Sequences from this database can contain near identical plasmids which could bias the downstream validation of mge-cluster. To select a single representative sequence among highly similar plasmids, we used cd-hit-est (version 4.8.1) to remove redundant sequences within a 0.99 sequence identity threshold (-*c* 0.99 -*s* 0.9 -*aL* 0.9) (23,24). Cd-hit-est generated 6185 groups encompassing plasmid sequences with high similarity and coverage, from these only a single representative sequence was chosen. The discarded sequences were used to benchmark the CPU time, runtime and memory required by mge-cluster to predict sequences considering an existing mge-cluster model. These sequences were also used as a quality check to ensure that mge-cluster returned the same cluster assignment as their cd-hit-est group.

We clustered the set of 6185 non-redundant plasmids using mge-cluster. The perplexity was set to 100 (*–perplexity*), with a minimum cluster size of 30 (*–min_cluster*). Unitigs were discarded if their variance exceeded 0.01 (*–variance*). We used the script average_nucleotide_identity.py included in the pyani package (version 0.2.11) to calculate the average coverage and average nucleotide identity (ANI) of the plasmids within each cluster (25). We performed distinct runs of mge-cluster setting distinct perplexity values (10, 30, 50, 200) to compare the resulting clustering solution against the presented mge-cluster model (perplexity = 100). For this, we considered the adjusted Rand index implemented in the function *adjustedRandIndex* from the mclust R package (version 5.4.7) (26). For representing the embedding created by openTSNE and the clusters defined by HDBSCAN, we used ggplot2 (version 3.3.6) and considered the Khachiyan algorithm implemented in the ggforce R package (https://github.com/thomasp85/ggforce) to draw ellipses around the clusters.

The clustering given by mge-cluster was compared against the current typing schemes: (i) ‘primary_cluster_id’ reported by the module MOB-typer of MOB-suite (12), a five-character fixed-length code that groups plasmids using complete-linkage clustering based on Mash distances (default distance = 0.06), (ii) plasmid taxonomic units (PTUs) reported by COPLA based on ANI distances and hierarchical stochastic block modelling (11) and (iii) *in silico* pMLST data retrieved from the PLSDB database, a typing scheme based on the combination of allelic variants present in well-known plasmid replicons (6). Due to the CPU time and memory required by COPLA to predict a single sample, we could not perform the typing and comparison of all the 6185 plasmid sequences included in the model. Instead, from these 6185 sequences, we considered 695 plasmids typed with a PTU in a recent publication introducing COPLA (10). The pMLST typing retrieved from the PLSDB database was based on six different schemes (IncA/C, IncF, IncHI1, IncHI2, IncI1 or IncN) and the typing provided in the PLSDB database was performed using a minimum identity of 85% and minimal coverage of 66% (22). In the case of plasmids annotated with the IncF scheme, we considered the replicons with multiple perfect hits against known allelic variants as a single type (e.g. FIA 1,6) and unambiguous hits (marked with ‘?’ in the PLSDB annotation) considered as an allelic variant missing (–). This created a FAB formula based on the replicons FIA, FIB, FIC, FII for which hits against these replicons were marked with the respective allelic variant and replicons with no hits typed as (–).

To quantify the concordance of the clustering solutions, we compared MOB-suite, COPLA and pMLST against mge-cluster considering the adjusted Rand index (26). This metric compares two clustering solutions for the same set of points and returns a value ranging from 0 (no similarity) to 1 (identical clustering). The pairwise comparisons were only performed with sequences with a defined cluster for any of the typing tools, thus discarding plasmids labelled as –1 for mge-cluster or with an unknown PTU (‘–’) by COPLA. In the case of pMLST, we discarded plasmid sequences with more than one Inc scheme annotated. To further inspect the level of concordance between typing schemes, for each mge-cluster we computed its Simpson diversity for replicon, MOB-suite clusters (‘primary_cluster_id’) and COPLA PTUs. For pMLST, we only considered the mge-clusters for which the majority of plasmids were annotated with a pMLST type (mge-clusters: 1, 6, 23, 24, 25, 26, 27, 28, 29, 30, 31, 32, 33, 34, 35, 36, 37, 38, 39, 40). We considered the function diversity implemented in the vegan R package (version 2.5–7) specifying the ‘simpson’ index. This value can range from 0 (no diversity, same clustering solution) to 1 (high diversity, distinct clustering solution). To illustrate the differences between the clusterings given by mge-cluster and MOB-suite, we performed a gene synteny analysis with clinker (version v0.0.21) (27) using two randomly chosen sequences belonging to the same mge-cluster but differing in their MOB-suite cluster. To visualize the diversity of clustering solutions within each mge-cluster, we used the treemapify R package (version 2.5.5) which produces treemaps for displaying nested and hierarchical data (https://github.com/wilkox/treemapify).

To assess the performance of mge-cluster assigning plasmid sequences with a distinct gene content and origin, we considered all plasmid sequences (n = 1020) with the species ‘*Staphylococcus aureus’* annotated from the PLSDB database and used the operational mode –*existing* of mge-cluster to assign these sequences to the clusters defined in the *E. coli* mge-cluster model. In the same manner, we typed all IncN plasmids (n = 206) from PLSDB belonging to a species different to *E. coli* annotated in the database and having uniquely a single replication gene in the field ‘PlasmidFinder’.

To illustrate the potential of mge-cluster to track the distribution of a gene-of-interest, we searched for AMR genes in our *E. coli* dataset using AMRFinderPlus (version 3.10.18) indicating as organism (-O) *Escherichia*, specifying the –*plus* flag and other default settings (28). From the resulting report, we searched for plasmid sequences encoding for the gene *mcr-1.1* (NCBI Reference Sequence accession NG_050417.1).

### Assessing the robustness of mge-cluster with ONT-simulated plasmids and simulated plasmid-predicted bins

To simulate plasmids obtained only with Oxford Nanopore Technologies (ONT), we considered the 5996 plasmids used by mge-cluster after removing plasmids with no unitigs which constitute the *E. coli* model presented in the manuscript (Supplementary Table S1). The simulation was performed with Mutation-Simulator (version 3.0.1) (29) which introduces random SNPs and insertion/deletions (indels) at a fixed rate. Based on our previous study (30), we showed that ONT-only assemblies had an average of ∼130 SNPs/100 kb and ∼140 indels/100 kb which was matched with the Mutation-Simulator arguments (-*sn* 0.0013, -*in* 0.00075, -*de* 0.00075). The shortest and longest length of the indels was 7 bp (-*inmin* 7) and 9 bp (-*inmax* 9), respectively, which corresponds to the most frequent homopolymer lengths wrongly called in ONT-only assemblies (http://albertsenlab.org/wp-content/uploads/2020/02/R10.3_dist_len_hp.pdf). Mge-cluster was run in the operational mode –*create* (–*perplexity* 100 –*min_cluster* 30) considering as input 11992 sequences (5996 original plasmids and 5996 ONT-simulated plasmids). In addition, mge-cluster was run in the operational mode –*existing* considering the *E. coli* model presented in this manuscript (https://doi.org/10.6084/m9.figshare.21674078.v1).

To simulate predicted plasmid bins, we selected 41 complete genomes from our previous study (30) where: (i) short-reads, (ii) ONT reads and (iii) Unicycler (hybrid) assemblies were all available. We extracted the short-read contigs from the best SPAdes graph considered by Unicycler (‘001_best_spades_graph.gfa’) and mapped them with Quast (v5.0.2) against each of the reference plasmids (*n* = 108) from these 41 complete genomes. Quast was run with the argument (–*min-contig* 1000) to map only contigs with a minimum size of 1 kb. Shorter contigs are neither frequently classified nor binned by plasmid prediction tools, thus their inclusion in the bins is not well justified. We used mge-cluster in the operational mode –*create* with a perplexity value of 5 (adjusted automatically by mge-cluster based on the sample size) and minimum cluster size of 2 (to allow each prediction to form an independent cluster with its associated reference plasmid).

For all cases, we assessed whether: (i) the reference plasmid and simulated plasmid/bin had the same mge-cluster assignment, (ii) the simulated plasmid/bin was unassigned but the reference plasmid belonged to a mge-cluster or (iii) the simulated plasmid/bin and reference plasmid belonged to distinct mge-clusters.

## RESULTS

### Generating a typing scheme for *Escherichia coli* plasmids

To evaluate the applicability and robustness of mge-cluster on real data, we generated a plasmid typing scheme for *E.coli* plasmids. We considered all plasmids from the curated PLSDB plasmid database (22,31) that includes samples from distinct isolation sources, hosts and countries. This dataset contained highly similar sequences that could lead to an overestimation of the performance of mge-cluster. Thus, redundant sequences were filtered using cd-hit-est (see Materials and Methods) to select a single representative plasmid among highly similar sequences (*n* = 6185). The discarded plasmid sequences (*n* = 675) were used as a further test set for benchmarking the runtime and memory required for mge-cluster.

**Figure 2.**
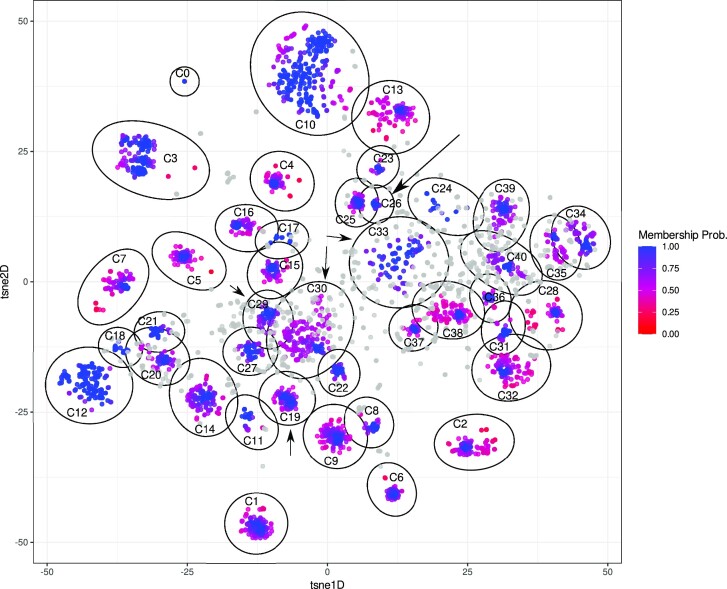
OpenTSNE embedding based on unitigs (*k* = 31) of 5996 *E. coli* plasmids. Each point corresponds to a plasmid sequence and their assigned cluster (C) is labelled based on the cluster ID (*n* = 41) defined by HDBSCAN. Sequences belonging to an HDBSCAN cluster are coloured (from red to blue) based on their membership probability. Unassigned sequences correspond to plasmids with a membership probability of 0 of belonging to any defined cluster and are coloured in grey. The ellipses (in black) delimit the cluster coordinates and were estimated using the Khachiyan algorithm implemented in the ggforce R package. To facilitate finding clusters 19, 26, 29, 30 and 33, which are highlighted as examples in the text, we indicated their positions with an arrow in the plot.

After removing uninformative unitigs (*k* = 31) with low variance (0.01, *n* = 680 491), mge-cluster considered 211198 unitigs as input to generate the classification framework. The resulting unitigs had an average size of 37.52 bp (median = 33.00 bp). This left 189 plasmids (3.1%, 189/6185) without unitigs and these plasmids were excluded from subsequent clustering analysis resulting in 5996 remaining plasmids in the analysis. The filtered unitig presence/absence matrix was embedded with openTSNE (perplexity = 100) and clusters were called using HDBSCAN (min_cluster = 30). In total, we obtained 41 discrete plasmid clusters grouping 4784 sequences (79.8%, 4784/5996) with 1212 sequences remaining unassigned (20.2%, 1212/5996) (Figure 2, Supplementary Table S1).

The chosen perplexity value can impact the non-linear resultant embedding such that low perplexity values tend to preserve the local structure better, while sometimes artificially introducing some structure when none exists. Conversely, high perplexity values tend to preserve more of the global structure at the cost of merging small clusters together. We evaluated the impact of varying this mge-cluster parameter (perplexity = 10, 30, 50, 200) by comparing their resulting clustering assignments using the adjusted Rand index. This index can vary from 0 (completely distinct typing models) to 1 (identical typing models) while adjusting for randomly assigning two sequences belonging to the same cluster. We observed that mge-cluster produced assignments robustly (average Rand index = 0.95) to the chosen perplexity values when considering sequences assigned by two resulting models (Table 1). In addition, we show that the mge-cluster discrepancy between models can be explained by the sequences which are unassigned by one of the models but clustered in the other (Table 1). Consequently, we encourage users to run mge-cluster by setting distinct perplexity values to evaluate cluster stability.

**Table 1.** Comparison of the mge-cluster models over a range of perplexity values (10, 30, 50, 200). The models were compared against the mge-cluster solution, corresponding to a perplexity value of 100. The Rand index was first computed considering only points assigned to a cluster by the two clustering solutions and thus ignoring points which were either unassigned by one of the two models. Secondly, the Rand index was computed with all points (assigned and unassigned) to highlight the discrepancy between the models is mainly caused by sequences clustered by one of the two models but unassigned by the other

mge-cluster perplexity	Assigned points	Unassigned points	Number of clusters	Rand index - only assigned points	Rand index - all points
10	3778	2218	45	0.90 (3,502)	0.29 (5,996)
30	5187	809	44	0.95 (4,651)	0.61 (5,996)
50	4887	1109	45	0.96 (4,579)	0.70 (5,996)
200	4528	1468	38	0.98 (4,392)	0.70 (5,996)

Plasmids can rapidly incorporate or lose genomic modules or even co-integrate with other sequences present in the same cell, which drastically affects their size. For each cluster (*n* = 41) (perplexity = 100, min_cluster = 30), the interquartile range (IQR) of the sequence length was on average 18.66 kb but with pronounced differences depending on the cluster (Supplementary Table S2). As an example, cluster 26 (Figure 2) with a mean length of 94.6 kb showed an IQR of 0.26 kb indicating an almost intact plasmid backbone, while, cluster 19 (Figure 2) with a mean length of 159.4 kb had an IQR of 51.2 kb indicating the presence of distinct gained/lost genomic modules shared by only a fraction of the plasmids assigned to this cluster.

To quantify the percentage of shared sequences among plasmids from the same cluster, we used pyani to retrieve average nucleotide identity (ANI) and coverage values (32). On average, plasmids shared 62.3% of their sequence (pyani coverage) with other members from the same cluster with an associated ANI of 95.7%. We observed that the average coverage shared between plasmids varied substantially among clusters indicating distinct degrees of plasmid modularity as previously exemplified with the IQR of the sequence length (Supplementary Table S2). Clusters 29, 30 and 33, formed by large plasmids, displayed a low pyani coverage indicating that plasmids from those clusters shared only a minor fraction of their sequence. To further understand the content of each mge-cluster, we visualized the diversity of replicons (Supplementary Figure S2) predicted by the MOB-typer module of MOB-suite (12) based largely on PlasmidFinder (6).

### Comparison of mge-cluster against other plasmid typing tools

To assess the level of concordance with current typing schemes, we compared the mge-cluster results against the gold standard methods for plasmid typing. MOB-suite provides a five-character fixed-length code (2 letters and 3 digits) to identify sequences belonging to the same group (termed ‘primary_cluster_id’) (Supplementary Table S3), while COPLA provides a PTU designation (Supplementary Table S4). However, the CPU time (167 minutes, 22 min wall-clock time) and memory (319.5 Mb) required for COPLA to predict the plasmid type of a single sequence (NZ_CP024805.1) hampered us from predicting the entire *E. coli* dataset of 6185 plasmids for a full comparison with mge-cluster. However, 695 sequences (11.2%, 695/6185) from our dataset were typed in the original publication describing COPLA [10] and were further considered in this comparison. In addition, we retrieved from the PLSDB database the information derived from *in silico* pMLST data (Supplementary Table S5). pMLST includes six different schemes to assign each of the replicons present in the plasmids with a distinct allelic variant. The resulting allelic variant combination (FAB formula in the case of IncF plasmids) is frequently reported in studies to group plasmids into different types.

**Figure 3.**
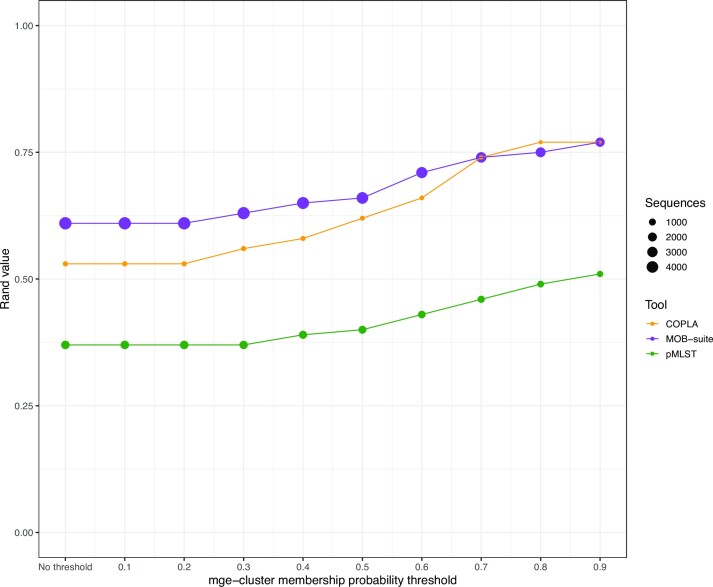
Concordance of mge-cluster results compared to MOB-suite (in purple), COPLA (in orange) and pMLST (in green) based on adjusted Rand index values. For each sequence assigned to a cluster by mge-cluster, the tool returns a membership probability. This probability was used to set several thresholds (ranging from no threshold to 0.9) to assign the plasmid sequences and assess their concordance against MOB-suite, COPLA and pMLST. Each point in the comparison is sized according to the number of sequences used to compute the adjusted Rand index value between the tools.

To compare the overall clustering concordance, we considered the adjusted Rand index which fluctuates from 0 (maximally different clustering) to 1 (identical clustering). We observed a moderate agreement between mge-cluster and MOB-suite with an index of 0.61, for COPLA the adjusted Rand index was 0.53 while pMLST showed the lowest level of agreement (0.37) (Figure 3, no threshold). Notably, we observed that increasing the membership probability threshold of mge-cluster to assign plasmids to particular clusters resulted in a higher level of overlap between the tools reaching a maximum adjusted Rand index value of 0.77 for MOB-suite and COPLA, and 0.51 for pMLST (Figure 3, threshold = 0.9).

To define which mge-clusters had a higher level of overlap with MOB-suite, COPLA and pMLST types, we calculated the Simpson diversity of each mge-cluster. For instance, if all plasmids from a particular mge-cluster were designated as a single type by MOB-suite, COPLA or pMLST, this Simpson diversity value would be 0. In contrast, the presence of multiple types defined by MOB-suite, COPLA or pMLST would result in diversity values close to 1. For pMLST, we only calculated the Simpson diversity of mge-clusters for which the majority of plasmids had a pMLST annotation since not all *E. coli* plasmids had an associated Inc scheme (see Materials and Methods). The diversity of MOB-suite and COPLA types is shown in Supplementary Figures S3 and S4, respectively.

**Figure 4.**
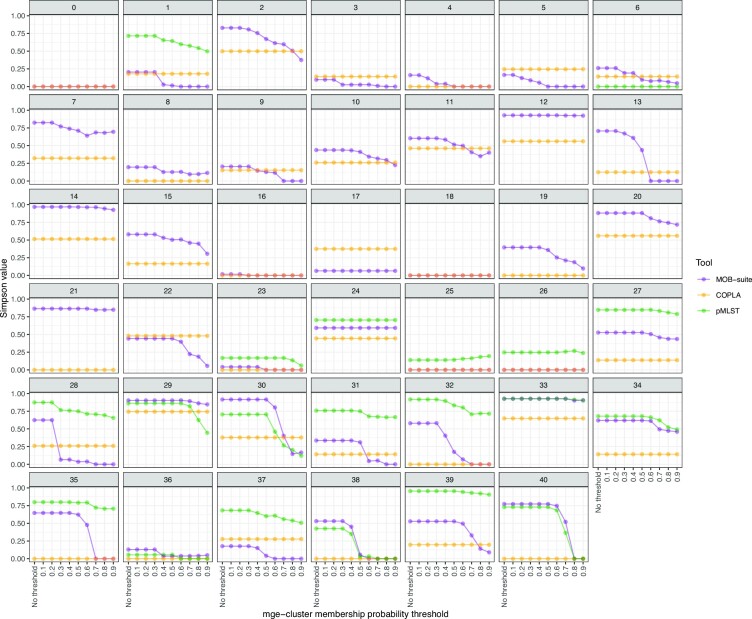
Diversity of MOB-suite (in purple), COPLA (in orange) and pMLST (in green) types for each mge-cluster (*n* = 41). The diversity (Simpson value) could range from 0 (agreement with mge-cluster) to 1 (no agreement). The probability assigned to each plasmid by mge-cluster was considered to set several thresholds (ranging from no threshold to 0.9) to assign the plasmid sequences and assess their concordance with MOB-suite, COPLA and pMLST.

The overall Simpson diversity per cluster was 0.61, 0.46 and 0.21 for pMLST, MOB-suite and COPLA, respectively. We observed that by increasing the membership probability threshold, the average diversity of MOB-suite types was substantially reduced up to 0.23 (threshold = 0.9), in the case of pMLST up to 0.43 (threshold = 0.9) with no changes in the case of COPLA (0.21, threshold = 0.9) (Figure 4).

We observed that mge-cluster grouped together plasmids belonging to the same Inc scheme but distinct pMLST type, exemplified by mge-clusters 1 (IncHI2 related) and 27 (IncN related). For the plasmids typed by pMLST according to the IncF RST scheme, we observed that mge-cluster frequently clustered together plasmids with a distinct FAB formula (33). For instance, mge-cluster 35 mostly consisted of plasmids with the FAB formula F2:A-:B- (e.g. AP024132.2, membership probability = 1.0) but also F24:A-:B1 (e.g. NC_011747.1, membership probability = 1.0). Blastn analyses between NC_011747.1 (F24:A-:B1) AP024132.2 (F2:A-:B-) and showed 92% query coverage and 99.93% sequence identity (NC_011747.1 query; AP024132.2 subject). In mge-cluster 39, plasmid sequences CP057140.1 (membership probability = 1.0) and CP057627.1 (membership probability = 1.0), which had a distinct FAB formula (F56:A-:B- and F89:A6:B-), were clustered together. Blast analyses (CP057140.1 query; CP057627.1 subject) revealed 98% query coverage and 99.92% sequence identity between these plasmids. This can be explained by a high rate of acquisition/loss of the replicons used to infer the FAB formula as well as the accumulation of mutations. These results illustrate that pMLST typing tends to only group together plasmid sequences exhibiting a very limited level of divergence. In contrast to the other tools considered here, pMLST is only able to type a subset of the *E. coli* plasmids since some sequences contain replicons for which a pMLST scheme is not available.

COPLA produced the same PTU designation (PTU-FE) for 10 distinct mge-clusters, which resulted in a lower Simpson diversity than for MOB-suite at the cost of merging together plasmids with a distinct core gene content (Supplementary Figure S4). This PTU-FE type was reported in the original COPLA article as problematic because several plasmid configurations were present resulting in a low intra-cluster density (10).

MOB-suite showed a consistent agreement (Simpson value < 0.2) in 13 mge-clusters (Figure 4, Supplementary Table S2). The disagreement occurred in the mge-clusters with an average small plasmid length (<10 kb) (clusters: 2, 7, 12, 14, 20, 21). These clusters consisted of sequences with a predominant replicon type (Supplementary Figure S2), however, MOB-suite predicted those sequences in distinct clusters (Supplementary Figure S3). MOB-suite confirmed with a high Simpson diversity value, that clusters 29, 30 and 33 were formed by large plasmids from distinct types (Supplementary Table S2).

For the remaining clusters we observed that mob-cluster tended to only group sequences that were highly similar in their gene content (high identity and coverage). To illustrate this, we considered a random sequence from mge-cluster 31 predicted with a different type by MOB-suite (NZ_LT985213.1 for AA735, NZ_CP010138.1 for AA334) and performed a gene synteny analysis (Supplementary Figure S5). We observed that these two sequences, despite being classified by MOB-suite as distinct types (AA735 and AA334), had a blastn coverage and identity of 73.1% and 99.6%, respectively. The synteny analysis revealed both sequences had an IncFII replicon with a well-conserved synteny (Supplementary Figure S5). However, NZ_LT985213.1 had incorporated an extra module corresponding to the co-integration of an IncFIA replicon. MOB-suite uses a stringent Mash threshold (0.06) to group plasmid sequences, and consequently, sequences that share a highly similar plasmid backbone, but have gained or lost genomic modules, or even co-integrated other plasmids, tend to be grouped by MOB-suite into distinct types. In the case of mge-cluster, plasmids acquiring an extra genomic module have a lower membership probability of belonging to the cluster since their unitig content differs, but are still considered to be part of the same cluster. This behaviour explains why the increase in the membership threshold of mge-cluster results in a higher agreement with MOB-suite (Figure 3).

### Predicting novel sequences with an existing mge-cluster model

Mge-cluster was built to generate a classification network that can also assign the same cluster names without the requirement to re-analyze any previous dataset and to keep consistent cluster names (*–existing* mode). We considered the sequences discarded by cd-hit-est (*n* = 675) to benchmark the runtime and memory required by mge-cluster to assign these sequences to the previous clusters. In addition, these sequences should be embedded and assigned to the same cluster as the representative sequence from the cd-hit-est step.

Mge-cluster predicted these 675 samples using less CPU and wall-clock time (23.3 min, ∼4 min wall-clock time) than for MOB-suite (CPU time 32.2 min, ∼ 26 min). However, the peak memory usage of mge-cluster (15.9 Gb) was substantially higher than for MOB-suite (4.5 Gb). From these 675 samples, 15 sequences corresponded to cd-hit clusters for which its representative sequence was discarded in the mge-cluster model because of the absence of unitigs and were not evaluated further. Mge-cluster correctly assigned 99.2% (655/660) of the plasmids to the same cluster as their corresponding reference sequence (Supplementary Figure S6). In five cases (0.8%, 5/660), mge-cluster predicted another cluster, including four cases where the model returned an unassignment (-1) category.

Next, we evaluated the performance of mge-cluster predicting plasmids not present in *E. coli* and thus unseen by the approach to build the mge-cluster model. For this, we considered all *Staphylococcus aureus* plasmids (*n* = 1020) from the PLSDB database because of the absence of plasmid transmission events between these two species (9,10). Mge-cluster did not detect any *E.coli*-specific unitigs (0 from a total of 211198 unitigs) for 972 *S. aureus* plasmids (95%) and thus those sequences were not assigned to any of the mge-clusters from the *E. coli* model (Supplementary Table S6). This is due to the high specificity of the unitigs used in the mge-cluster model which had a minimum size of 31 bp and an average size of 37.52 bp. From the remaining 49 plasmids (5%), 28 plasmids were not assigned to any cluster, 12 plasmids were assigned to the mge-cluster 29 and 8 plasmids to the mge-cluster 30. We confirmed that the plasmids assigned to mge-clusters 29 and 30, had a low number of unitigs present and thus corresponded to samples with a vector of nearly all zeros. In those cases, mge-cluster embedded those sequences into clusters 29 and 30 which we previously highlighted as random noise clusters.

Lastly, we assessed the performance of mge-cluster predicting plasmids likely shared in other bacterial species from the same family (*Enterobacterales*) as *E. coli*. For this, we selected plasmids from the incompatibility group N (IncN) since they have a conserved core genome, which was used to develop a specific pMLST scheme (6) and have been reported across several bacterial species belonging to *Enterobacterales (**34**)*. We considered all IncN non-*E.coli* plasmids from the PLSDB database containing uniquely a single replication gene (*n* = 206) and predicted their clustering assignment with the *E. coli* mge-cluster model (Supplementary Table S7). We observed that most IncN plasmids (80.6%, 166/206) were predicted as part of the mge-cluster 27 which contains a majority of *E. coli* plasmids belonging to this incompatibility group (Supplementary Figure S2) and thus confirming that this plasmid type is shared and has a conserved genomic backbone among *Enterobacterales*. In total, 34 plasmids (16.5%) could not be assigned to any mge-cluster and were labelled as (–1) showing that some of these IncN plasmids might have acquired or recombined with other genomic modules and thus have a clearly distinct unitig content. The remaining plasmids (2.9%, 6/206) were scattered among mge-clusters 29 (*n* = 4), 14 (*n* = 1) and 30 (*n* = 1).

### Mge-cluster is robust to both long-read and short-read plasmid assemblies

The plasmid typing scheme provided by mge-cluster was built based on complete plasmid sequences from the PLSDB database. To obtain complete plasmid sequences, ONT sequencing is widely used. However, assemblies based only on ONT reads can contain SNPs, and in particular insertions and deletions (indels), often introducing errors and false premature stop codons that would affect protein predictions (35).

To estimate the robustness of mge-cluster to ONT-only plasmid assemblies, we randomly introduced SNPs and indels in the 5996 plasmid sequences considered as input for the presented *E. coli* plasmid typing scheme. The rate of SNPs and indels (see Materials and Methods) present in ONT-only based plasmids was fixed to simulate ∼130 SNPs/100 kb and ∼140 indels/100 kb based on our previous study (30). To assess if the reference and ONT-simulated plasmids would cluster together, we used the two operational modes of mge-cluster with a perplexity value of 100 and minimum cluster size of 2 in the –*create* mode. This minimum cluster size was determined so each pair of reference and ONT-plasmid could constitute an independent cluster. For both operational modes, we observed that ∼98% of the ONT-simulated plasmids were clustering together with their associated reference plasmids (Supplementary Figures S7A, S7B and Supplementary Tables S8, S9). In ∼2% of cases, the ONT-simulated plasmids were either unassigned or incorrectly grouped into a distinct mge-cluster.

Despite the growing number of complete plasmid sequences and the adoption of long-read sequencing technologies, short-read sequencing still remains the preferred technology in many microbiological laboratories for performing routine whole-genome sequencing. To estimate the robustness of mge-cluster clustering predicted-plasmid short-read contigs and complete plasmid sequences, we considered 41 *E. coli* complete genomes (108 plasmids) from our previous study (30) (Supplementary Table S10). Short-read contigs were extracted and mapped against the 108 reference plasmids to create bins with the unsorted short-read contigs. To simulate the output of plasmid prediction tools, we only mapped and included in the bins, short-read contigs with a length larger than 1 kb. Mge-cluster was run with the –*create* operational mode (–*perplexity* 5, –*min_cluster* 2) considering the 216 samples (108 reference plasmids, 108 short-read bins). In ∼97% of the cases, the short-read bin was grouped in the same cluster together with its associated reference plasmid (Supplementary Figure S7C). This is in line with the results based on ONT-simulated plasmids (Supplementary Figure S7), indicating the robustness of mge-cluster to both the sequencing technology and the completeness of the assembly.

### Showcasing mge-cluster in a real epidemiological study

To elucidate if the typing scheme provided by mge-cluster could be useful to infer plasmid transmission, e.g. in clinical settings, we considered a prospective observational cohort study (36) which involved patients admitted to two haematology wards at the Cambridge University Hospitals NHS Foundation Trust in England. In this study, the authors performed long-read sequencing of a representative set of *E. coli* samples and assessed if there was plasmid transmission among patients using genomic and epidemiological data. In total, the authors considered 16 complete plasmid sequences carrying the beta-lactamases genes *bla*_CTX-M-15_ or *bla*_CTX-M-14_ (Supplementary Table S11).

Mge-cluster grouped these 16 plasmids into 4 distinct clusters (Supplementary Figure S8, Supplementary Table S11). These clusters consisted of plasmids present in distinct *E. coli* sequence types (ST) but also plasmids present in different patients (Supplementary Figure S8). Ludden *et al.* found that the plasmids carrying the *bla*_CTX-M-15_ gene were variable in their replicon diversity but also in their antibiotic resistance gene content (36). Mge-cluster grouped the *bla*_CTX-M-15_ plasmids into three distinct clusters (mge-clusters 1, 2, 3), including a group corresponding to a bacteriophage-like plasmid (LR595875, LR595878 and LR595890). Based on epidemiological links, the authors discarded the horizontal spread among patients of any of the *bla*_CTX-M-15_ plasmids present in the study cohort.

The authors argued in the study that all *bla*_CTX-M-14_ plasmids belonged to the incompatibility group (IncB/O/K/Z) and mge-cluster grouped them into a single cluster (mge-cluster 0). These *bla*_CTX-M-14_ plasmids were retrieved from two patients (Supplementary Figure S8) and had a high pairwise blastn coverage (>90%) and identity (>98%) confirming the assignment given by mge-cluster. To determine if plasmid transmission might have occurred between the two patients, Ludden et. al performed a SNP analysis based on the plasmid core genome (36). They observed that the plasmids present in the two patients differed by hundreds of SNPs, thus discarding a plasmid transmission scenario.

Based on these results, we suggest that mge-cluster can be utilised as a first step to broadly cluster the plasmid sequences derived from epidemiological studies. However, each of the mge-clusters needs to be further inspected using a more refined SNP analysis based on the plasmid core genome, and epidemiological data should preferably be added to better assess direct plasmid transmission scenarios.

### Cluster distribution and visualization of a gene of interest in the embedding space

The typing scheme offered by mge-cluster is optimal for visualizing the genomes carrying any particular gene of special interest and tracking its distribution in future sequencing studies. To illustrate this, we considered the AMR gene *mcr-1.1* which confers resistance to colistin, a last-resort antibiotic for treating infections caused by multi-drug resistant *E. coli*. This AMR gene was first reported in 2016 on a plasmid with an IncI2-type backbone (37) that can be mobilised among distinct MGEs by the presence of an *ISApl1* transposon element situated upstream of the gene (38).

**Figure 5.**
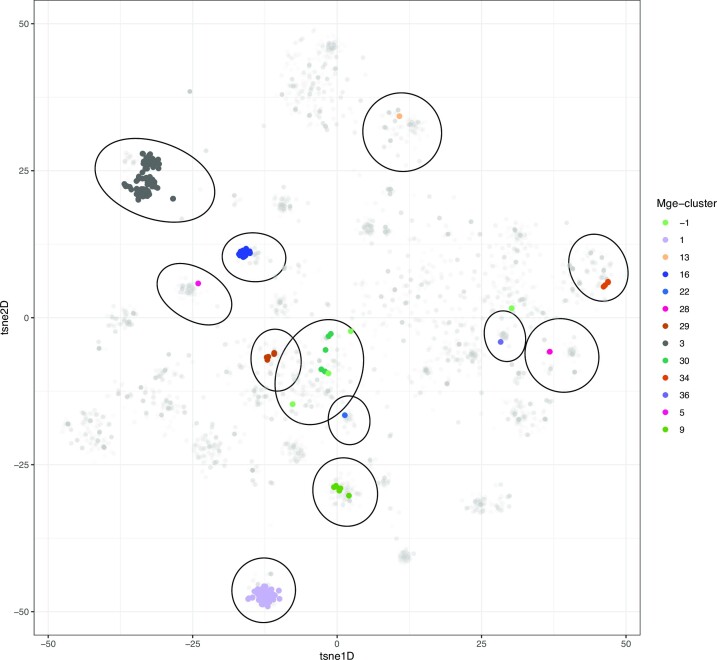
Distribution of the *mcr-1.1* gene on the embedding space created by mge-cluster. The plasmids (*n* = 327) containing the gene are coloured in the plot according to their cluster labels. The clusters (*n* = 12) containing at least a single sequence having the *mcr-1.1* gene are indicated with an ellipse using the Khachiyan algorithm implemented in the ggforce R package. The sequences which were not assigned to any mge-cluster were labelled as ‘–1’ (light green) in the legend.

We observed that 327 plasmids contained the *mcr-1.1* gene, the vast majority of these present in only three mge-clusters: 3 (*n* = 168), 1 (*n* = 71) and 16 (*n* = 53) (Figure 5). This was in agreement with previous reports (39,40) showing this AMR gene to be mainly spread by the plasmid backbones IncI2 (mge-cluster 3), IncHI2 (mge-cluster 1) and IncX4 (mge-cluster 16) (Supplementary Figure S2). However, we also observed that the AMR gene was present in nine additional mge-clusters (30/327, 9.2%) (Figure 5) and 5 sequences (1.5%) could not be assigned to any mge-cluster. This illustrates how a consistent typing provided by mge-cluster can be used to explore whether these nine clusters represent spillover events of the gene to other plasmid backbones for which the gene might be further disseminated using new plasmid types.

## DISCUSSION

The number of MGEs available in public databases has grown rapidly since the introduction of long-read sequencing technologies. However, the contextualization and comparison of MGEs are hampered by their high rates of recombination which result in the absence of a conserved marker that can be broadly used by standard phylogenetic methods. Mge-cluster responds to this need by generating discrete clusters from sequences generally evolving through a fast and dynamic turnover of gene gain/loss events.

We demonstrated the potential of mge-cluster by developing an *E. coli* model to classify plasmid sequences. We observed that the clusters generated by mge-cluster typically consisted of sequences with a shared plasmid backbone (coverage ∼62%) but distinct accessory content. Mge-cluster and MOB-suite showed a moderate level of agreement between clustering solutions. Some of the disagreement between the tools is explained by mge-cluster grouping together plasmid sequences that have acquired an extra replicon sequence due to the cointegration of another plasmid. This characteristic of mge-cluster is particularly beneficial for tracking a plasmid in the context of longitudinal studies for which the same plasmid can rapidly gain/lose genomic modules. The current version of COPLA makes the typing of large collections unfeasible due to the CPU time required to run a single sample. Moreover, in the particular case of *E. coli*, COPLA erroneously merges clusters from distinct plasmids under the PTU-FE group. In contrast to MOB-suite and COPLA, mge-cluster does not require a predefined distance threshold to generate the typing model which facilitates broad applicability across distinct species and datasets. pMLST, which is based on the assignment of allelic variants in well-known replicon sequences, showed the lowest agreement with mge-cluster. We demonstrated that in several examples, plasmids grouped together by mge-cluster were annotated with distinct pMLST types but had a high blastn coverage and identity. The rapid dynamics of acquisition/loss of replicons together with the incorporation of mutations in those replicon sequences limit the use of pMLST typing for comparing plasmids with limited evolutionary divergence.

Mge-cluster can be used to provide a typing scheme that can be easily shared and reused by other researchers. For generating a typing scheme, we recommend including only complete, and curated plasmid sequences similar to those provided in the PLSDB database (22,31). To avoid high levels of redundancy in the initial dataset, a filtering step with cd-hit-est (23) or MMseqs2 (41) is desirable to select only a single representative sequence among almost identical plasmids (e.g. plasmids sequenced from an outbreak scenario or longitudinal studies). We show that the clustering provided by mge-cluster is robust to the accuracy of long-read technologies. However, to generate a typing scheme, we believe that the inclusion of circular or true linear plasmids is desirable in the –*create* operational mode. Sequences which are not complete can be typed using the –*existing* operational mode. We show that mge-cluster can be used in other scenarios, such as for the validation and typing of plasmid-predicted bins based on short-read contigs (12,42,43). Furthermore, mge-cluster can be used to corroborate the predictions of a given tool (bin and reference plasmid cluster together) as well as to contextualize predicted plasmid bins with existing complete plasmid sequences (e.g. from the PLSDB database).

For epidemiological purposes, clusters obtained with mge-cluster should be interpreted in a similar manner as MLST (44) or BAPS groups (45) that cluster strains based on chromosomal housekeeping gene alleles and genome alignments respectively. Even if two plasmids from different patients belong to the same cluster, a direct plasmid transmission scenario cannot be automatically assumed. For this, mge-cluster can be considered as a starting point to perform secondary analyses, such as constructing SNP phylogeny based on the resulting cluster core genome. These secondary analyses can confirm or dismiss transmission links, as recently illustrated in two studies presented by Ludden *et al.* and Hawkey *et al.* (36,46). In the case of the study of Ludden *et al.*, we show that mge-cluster was useful to broadly type the plasmids into discrete groups, which opens the possibility of sharing the models for further tracking the distribution of a plasmid or gene-of-interest. In this case, we suggest that pMLST can be a valid and useful typing option since it provides a higher level of resolution than mge-cluster and plasmids involved in recent HGT events should have the same pMLST type.

While we demonstrated the use of mge-cluster using a single species, our approach can also be run on more diverse datasets such as the combination of plasmid sequences from the *Enterobacterales* family. We anticipate that mge-cluster can, in addition, be used for generating discrete clusters from other types of MGEs with sufficient gene content diversity including phages, integrative and conjugative elements (ICEs) or flanking sequences surrounding a gene-of-interest (e.g AMR genes).

The ability of mge-cluster to rapidly assign new plasmids with a consistent type facilitates the comparison of plasmids derived from distinct collections and boosts our capacity to conduct MGE surveillance in general.

## DATA AVAILABILITY

The mge-cluster package can be installed from bioconda https://anaconda.org/bioconda/mge-cluster under the open-source MIT license. Extensive documentation on mge-cluster usage is available at https://gitlab.com/sirarredondo/mge-cluster.

The code required to reproduce the results and figures presented in this manuscript is available as a Rmarkdown document at https://gitlab.com/sirarredondo/mge-cluster_manuscript.

The plasmid sequences retrieved from the PLSDB database used to generate the *E. coli* mge-cluster for plasmid classification are publicly available at NCBI and their accession numbers listed on Supplementary Table S1. The accession numbers from the *S. aureus* and non-*E.coli* IncN plasmids retrieved from the PLSDB database and considered to assess the performance of mge-cluster typing new MGE data are available in Supplementary Tables S6 and S7 respectively. The complete plasmid sequences considered derived from Arredondo Alonso *et al.* 2021 (30) and the simulated short-read bins are available as a figshare item at https://doi.org/10.6084/m9.figshare.23294909. The accession numbers from the complete plasmid sequences derived from Ludden *et al.* 2021 (36) are available in Supplementary Table S11.

The *E. coli* mge–cluster model presented in this manuscript is available as a figshare item at https://doi.org/10.6084/m9.figshare.21674078.v1.

## Supplementary Material

lqad066_Supplemental_Files
