# Supplementary material for: Mge-cluster: a reference-free approach for typing bacterial plasmids
Source: NAR Genom Bioinform. 2023 Jul 10;5(3):lqad066. doi: 10.1093/nargab/lqad066 (PMC10331934; doi:10.1093/nargab/lqad066)
Supplement: lqad066_Supplemental_Files [file lqad066_supplemental_files.zip › Legends_Supplementary_Tables.docx]

**Supplementary Table S1:** Mge-cluster results using the ‘create’ operational mode (perplexity = 100, min_cluster = 30) on the 5,996 plasmids derived from the PLSDB database and used to define the *E. coli* plasmid typing model. From the initial 6,185 non-redundant *E. coli* plasmid sequences, 189 plasmids were discarded by mge-cluster after removing unitigs with low variance.

**Supplementary Table S2:** General statistics on the 41 mge-clusters presented in the *E. coli* model. The average (mean) pyani pairwise coverage, pyani pairwise identity are indicated. For each tool benchmarked (MOB-suite, COPLA, pMLST) and mge-cluster, we present their corresponding Simpson diversity values.

**Supplementary Table S3:** MOB-suite results on the 6,185 non-redundant *E. coli* plasmid sequences derived from the PLSDB database.

**Supplementary Table S4:** COPLA results on the plasmid sequences used and reported in the publication by Redondo-Salvo *et al.* 2020 https://doi.org/10.1038/s41467-020-17278-2. From these sequences, 695 plasmids were also typed by mge-cluster and considered to do the comparison between mge-cluster and COPLA.

**Supplementary Table S5:** pMLST information processed from the PLSDB metadata.

**Supplementary Table S6:** Evaluation of mge-cluster assigning plasmids from a distinct bacterial species using the *E. coli* typing model. Mge-cluster was run with the ‘existing’ operational mode considering as input 1,020 *S. aureus* plasmids from the PLSDB database.

**Supplementary Table S7:** Evaluation of mge-cluster assigning plasmids putatively shared in other bacterial species present in the dataset used to define the *E. coli* typing model. Mge-cluster was run with the ‘existing’ operational mode using as input 206 IncN plasmids (pMLST scheme) belonging to *Enterobacterales* but from a distinct bacterial species than *E. coli*.

**Supplementary Table S8:** Evaluation of mge-cluster grouping together complete and polished plasmid sequences and ONT-only plasmid sequences using the ‘existing’ operational mode. The 5,996 plasmids used to define the *E. coli* model were mutated to introduce random SNPs and indels (size from 7 to 9bp) at a fixed rate simulating an average of ~130 SNPs/100kbp and ~140 indels/100kbp. Mge-cluster was run using the ‘existing’ operational mode considering as input the 5,996 ONT-simulated plasmid sequences and using the *E. coli* typing model presented. The columns starting with the prefix ‘original’ indicate the results given in Supplementary Table S1, while the columns starting with the prefix ‘predicted’ indicate the mge-cluster results on the 5,996 ONT-simulated plasmid sequences.

**Supplementary Table S9:** Evaluation of mge-cluster grouping together complete and polished plasmid sequences and ONT-only plasmid sequences using the ‘create’ operational mode. The 5,996 plasmids used to define the E. coli model were mutated to introduce random SNPs and indels (size from 7 to 9bp) at a fixed rate simulating an average of ~130 SNPs/100kbp and ~140 indels/100kbp. Mge-cluster was run with the ‘create’ operational mode (perplexity = 100, min_cluster = 30) considering as input 11,992 sequences (5,996 original plasmids and 5,996 ONT-simulated plasmids). Columns starting with the prefix ‘ref’ indicate the results of the sequences derived from the PLSDB database and columns starting with the prefix ‘sim’ indicate the results on the ONT-only simulated plasmids.

**Supplementary Table S10:** Evaluation of mge-cluster grouping simulated plasmid predicted bins and complete plasmid sequences using the ‘create’ operational mode. We considered 108 reference plasmids and simulated for each one a plasmid predicted bin. Mge-cluster was run in the ‘create’ operational mode (perplexity = 5, min_cluster = 2) with the 216 sequences (108 simulated plasmid bins, 108 reference plasmids).

**Supplementary Table S11:** Evaluation of mge-cluster on a real epidemiological study considering 16 plasmid sequences presented by Ludden *et al.* 2021. Mge-cluster was run in the ‘create’ operational mode (perplexity = 5, min_cluster = 2) with these 16 plasmid sequences. Information regarding the patient name and chromosomal sequence type (ST) was extracted from Ludden *et al.* 2021 https://doi.org/10.1016/S2666-5247(21)00117-8.
