# Supplementary material for: Mge-cluster: a reference-free approach for typing bacterial plasmids
Source: NAR Genom Bioinform. 2023 Jul 10;5(3):lqad066. doi: 10.1093/nargab/lqad066 (PMC10331934; doi:10.1093/nargab/lqad066)
Supplement: lqad066_Supplemental_Files [file lqad066_supplemental_files.zip › Revisions_NAR_GAB_Supplementary_Figures.pdf]

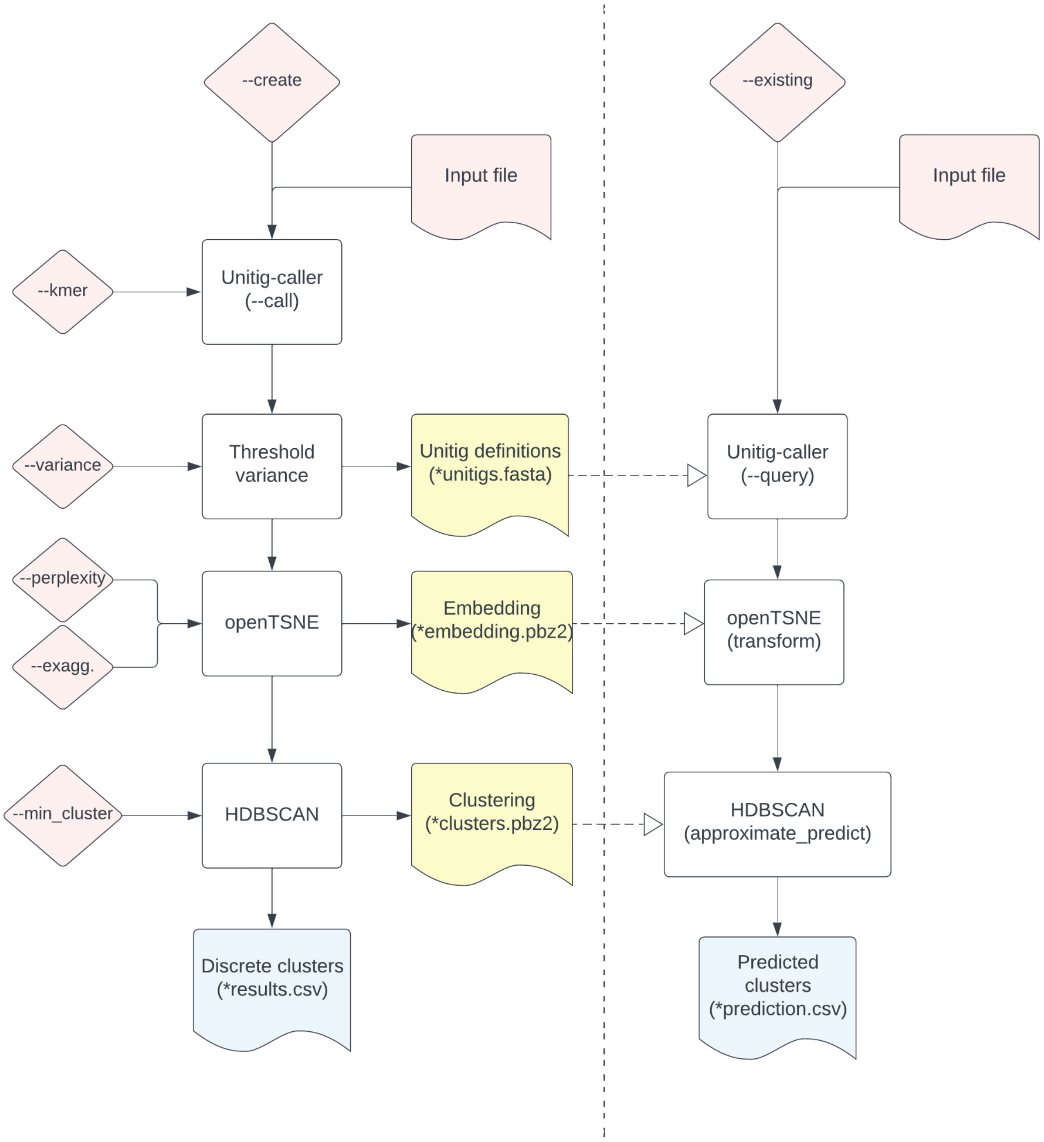

Supplementary Figure S1. Summary of the mge-cluster workflow. The tool is composed of two distinct operational modes: *--create* (left) and *--existing* (right). Both modes require as input a file listing the absolute or relative paths of the nucleotide sequences. The *--create* mode of mge-cluster requires the following arguments (*--kmer*, *--variance*, *--perplexity*, *--exaggeration* and *--min_cluster*) to generate discrete clusters from the sequences provided in the input. The *--existing* mode of mge-cluster requires the files (in yellow) generated by the *--create* mode to predict the clusters of a new batch of nucleotide sequences.

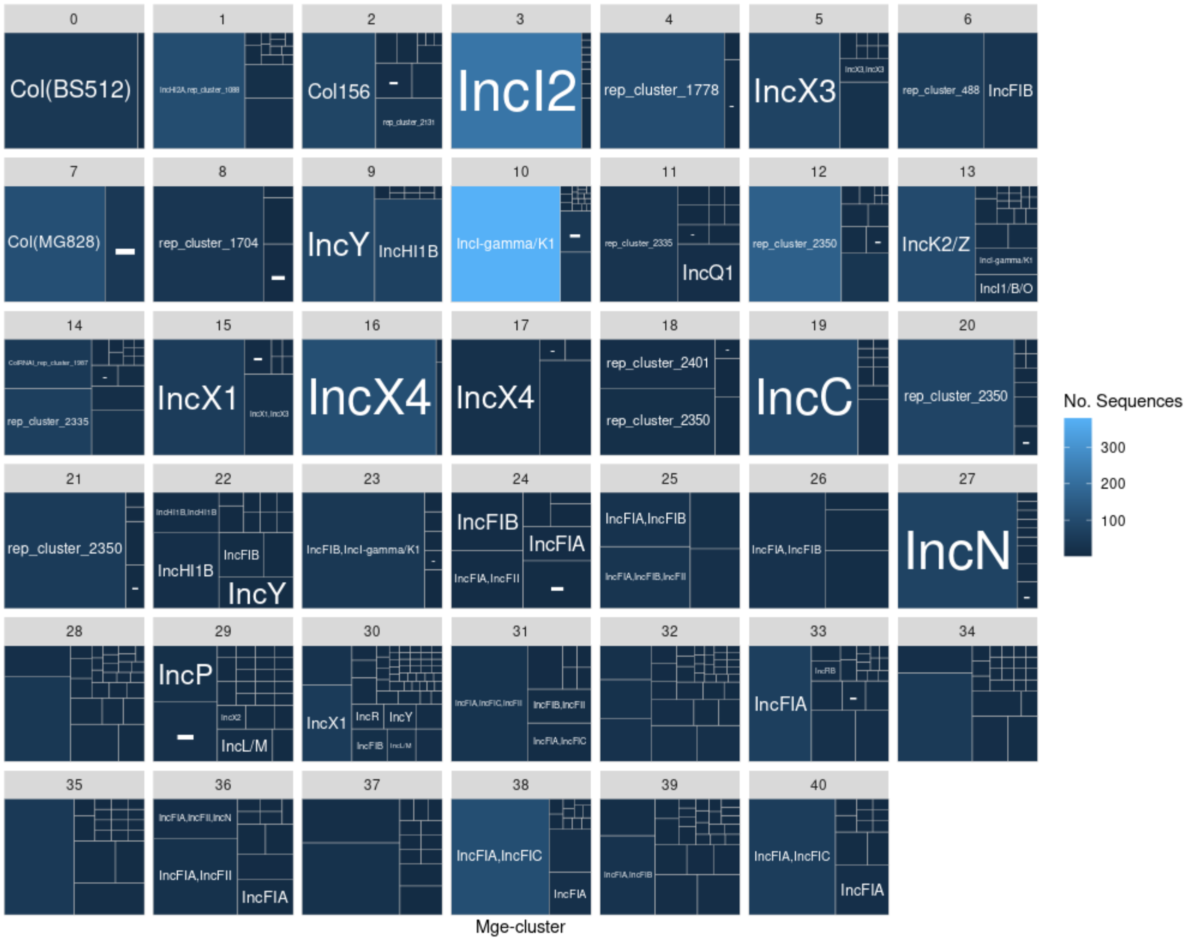

Supplementary Figure S2. Replicon diversity reported by the module MOB-typer of MOB-suite in each mge-cluster (n=41). For each mge-cluster, the area of the plot is proportionally split into distinct tiles based on the number of plasmids with the same replicon combination. For each tile, the replicon combination is indicated in the center. In some cases, the tile may not contain any text because (i) the replicon combinations are rare resulting on a small tile where the text indicating the replicon(s) present cannot be fitted or (ii) multiple replicons are present in the plasmid resulting on a long text that surpasses the area of the tile.

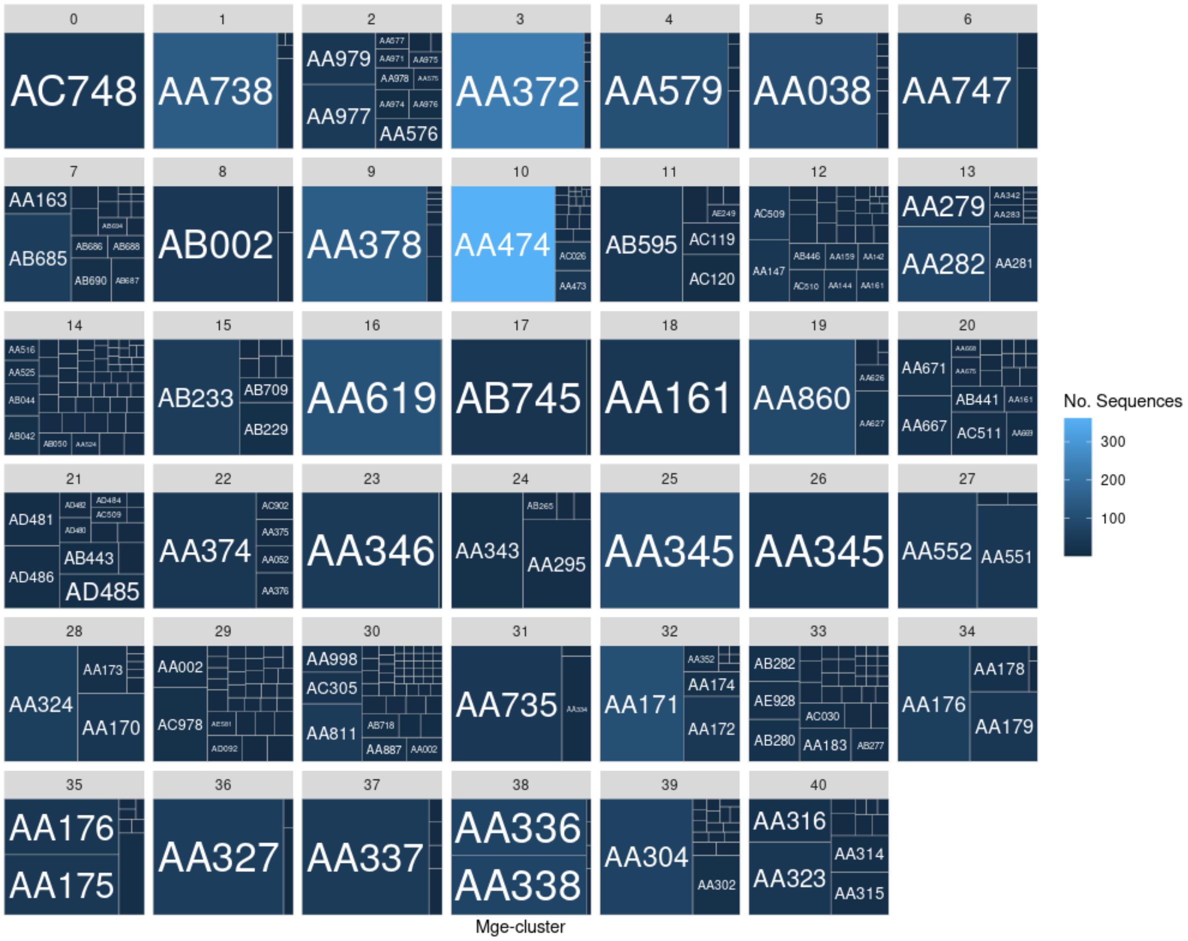

Supplementary Figure S3. MOB-suite cluster diversity (‘primary_cluster_id’) present in each mge-cluster (n=41). For each mge-cluster, the area of the plot is proportionally split into distinct tiles based on the number of plasmids with the MOB-suite plasmid type. For each tile, the MOB-suite type is indicated in the center. In some cases, the tile may not contain any text because the MOB-suite type is rare among the mge-cluster resulting in a small area where the text cannot be fitted.

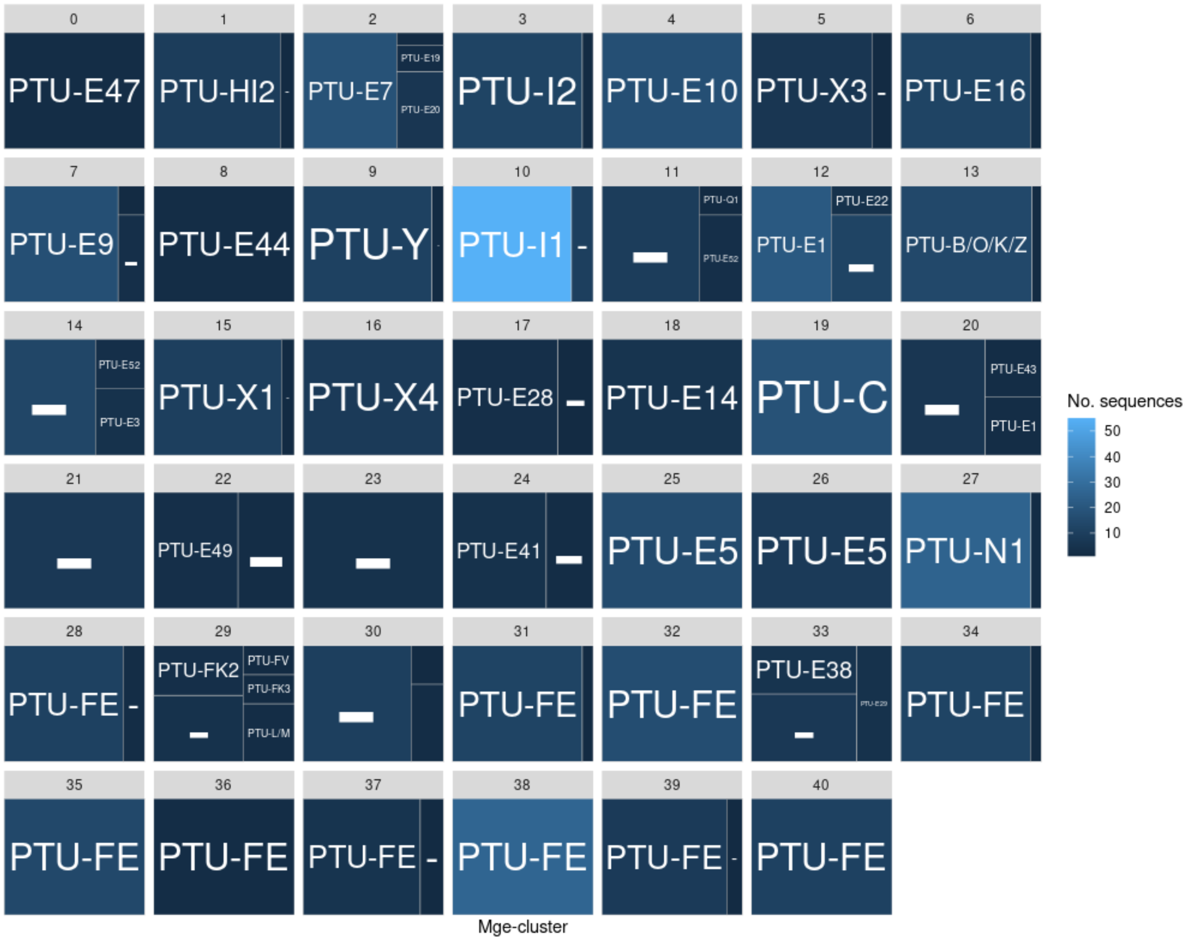

Supplementary Figure S4. COPLA plasmid taxonomic unit (PTU) diversity present in each mge-cluster. For each mge-cluster, the area of the plot is proportionally split into distinct tiles based on the number of plasmids with a particular COPLA PTU. This diversity is uniquely based on 695 plasmid sequences previously typed in COPLA's original publication [10]. In some cases, COPLA labelled sequences as '-' corresponding to plasmids with an unknown PTU.

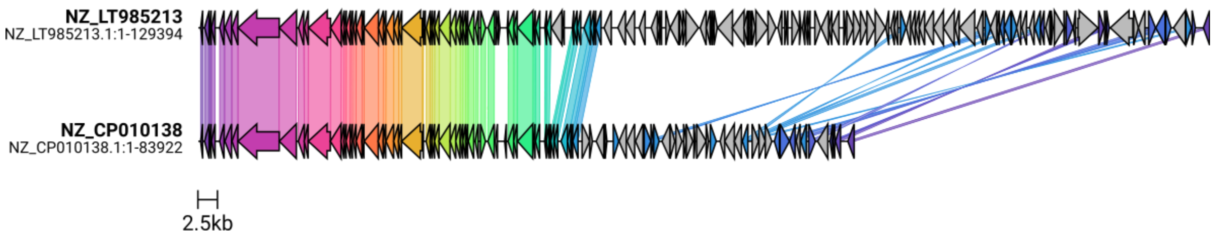

Supplementary Figure S5. Gene synteny plot between the sequences NZ_LT985213.1 (top) NZ_CP010138.1 (bottom) belonging to mge-cluster 31. These two sequences were randomly picked and represented the two major types (AA735 and AA334, respectively) defined by MOB-suite. The plot was created with clinker [41] based on the genome annotation (.gbk) computed with prokka [44], and homologous genes with a minimum identity of 80% are indicated with a link between the two sequences.

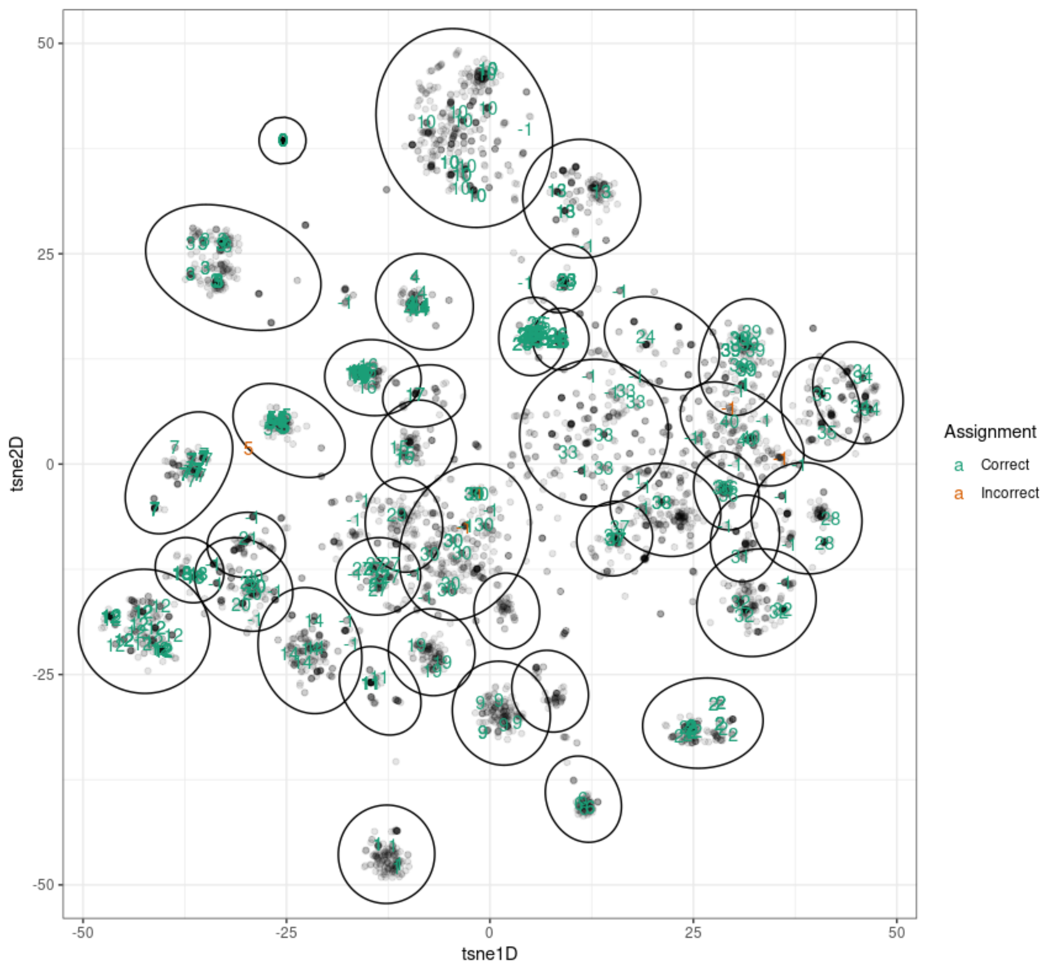

Supplementary Figure S6. Embedding and assignment of the plasmid sequences (n=675) that were originally discarded by cd-hist-est, and considered as a benchmarking set. These sequences are labelled based on their predicted HDBSCAN cluster and coloured based on whether their assignment was correct (in green) or incorrect (in orange).

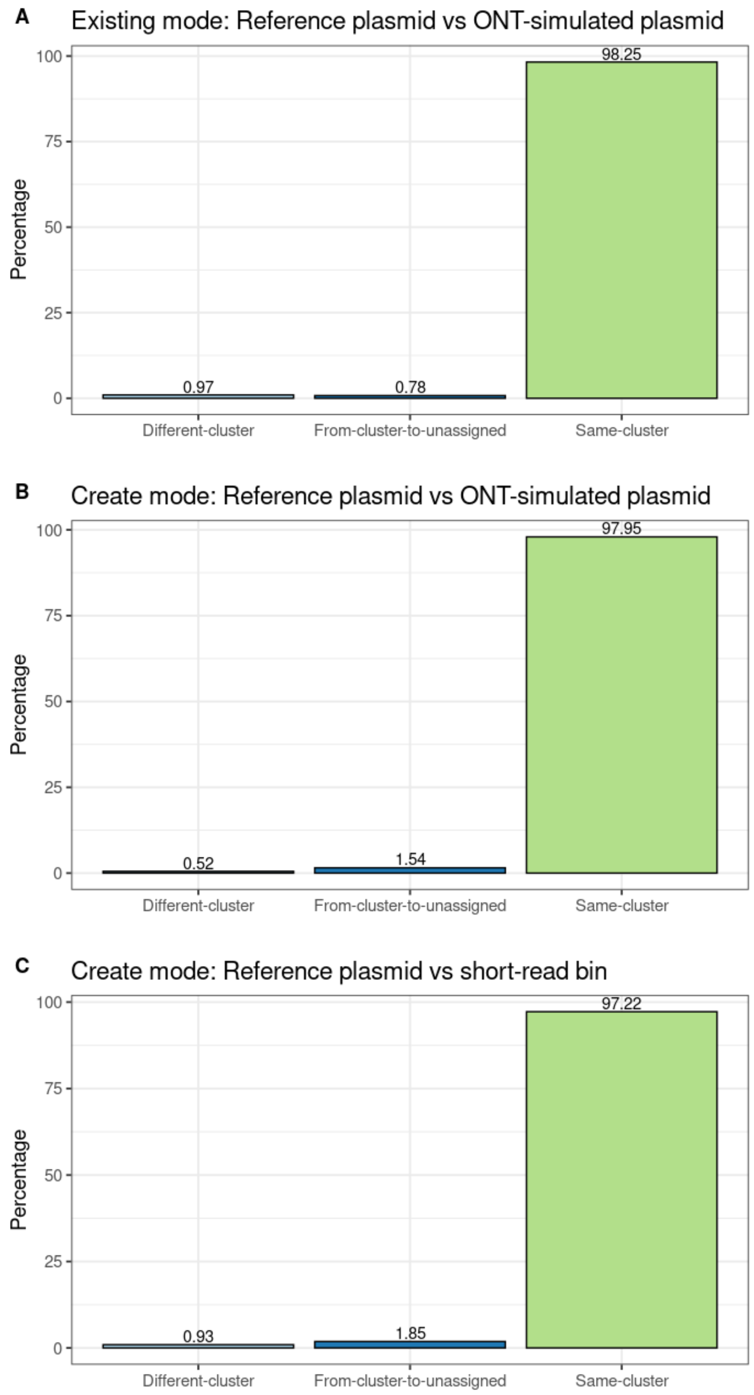

Supplementary Figure S7. Robustness of mge-cluster grouping sequences with a distinct degree of completeness. For each reference plasmid, we assessed whether its associated simulated sequence was assigned: i) with the same mge-cluster (Same-cluster bar), ii) unassigned by mge-cluster (From-cluster-to-unassigned bar) and iii) different mge-cluster (Different-cluster bar) **A)** The existing mode of mge-cluster was used to predict the embedding of 5,996 ONT-simulated plasmid sequences. **B)** The create mode of mge-cluster was used to cluster reference plasmid sequences (n=5,996) together with ONT-simulated plasmid sequences (n=5,996). **C)** The create mode of mge-cluster was employed to assess whether simulated predicted plasmid bins (n=108) clustered together with 108 reference plasmids described by Arredondo Alonso et. al 2021.

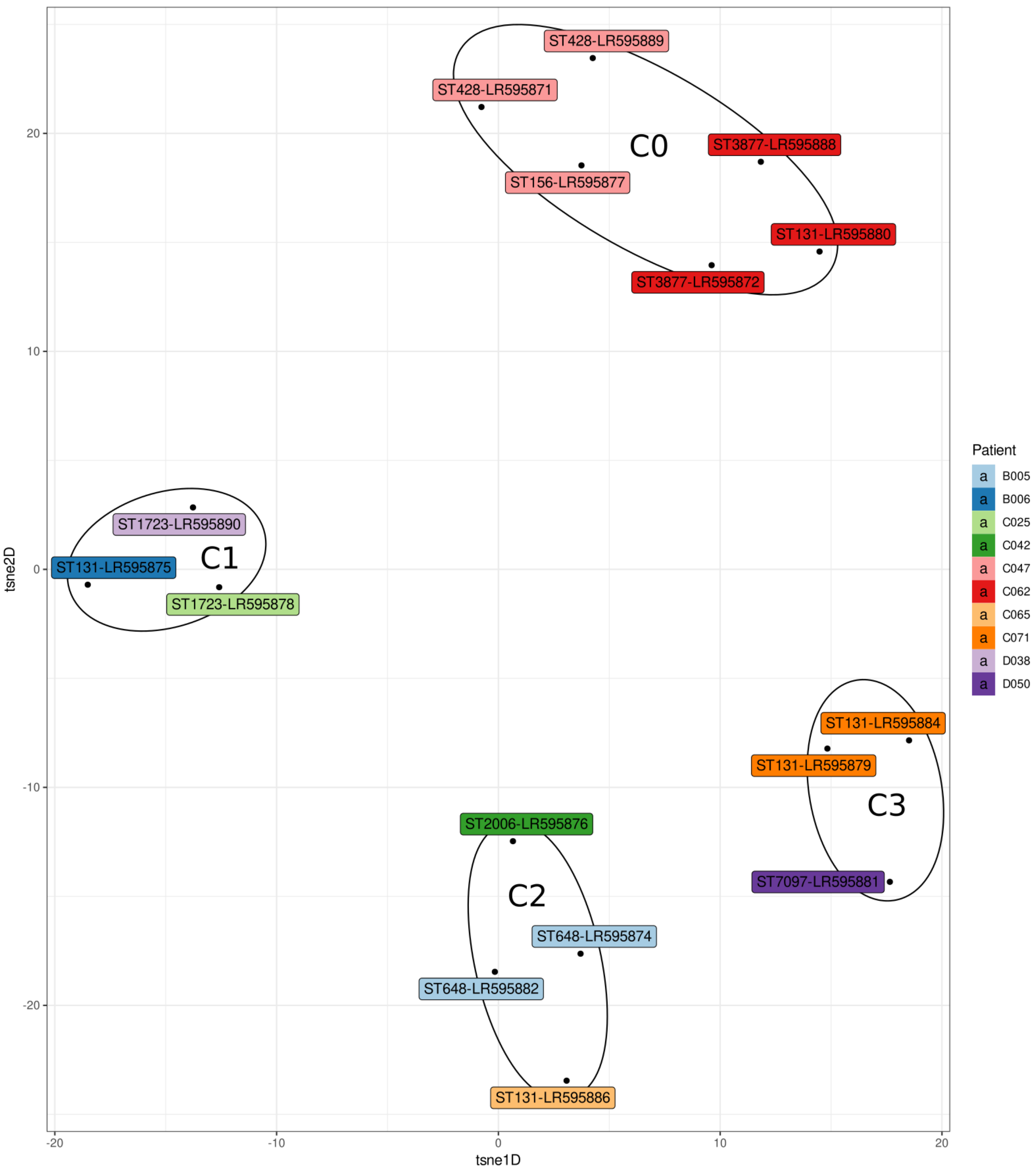

Supplementary Figure S8. Showcasing mge-cluster with the complete plasmid sequences (n=16) derived from the epidemiological study presented by Ludden et al. 2021. Each plasmid sequence was labelled with its corresponding chromosomal sequence type (ST) together with its accession number, and coloured accordingly to the patient carrying the plasmid. Each mge-cluster is indicated with an ellipse and its number labelled (C0, C1, C2 and C3).
